# Pubertal timing and breast density in young women: a prospective cohort study

**DOI:** 10.1186/s13058-019-1209-x

**Published:** 2019-11-14

**Authors:** Lauren C. Houghton, Seungyoun Jung, Rebecca Troisi, Erin S. LeBlanc, Linda G. Snetselaar, Nola M. Hylton, Catherine Klifa, Linda Van Horn, Kenneth Paris, John A. Shepherd, Robert N. Hoover, Joanne F. Dorgan

**Affiliations:** 1 Department of Epidemiology, Columbia University Mailman School of Public Health, 722 West 168th Street, Room 706, New York, NY USA; 2 Epidemiology & Public Health, University of Maryland School of Medicine, Baltimore, MD USA; 3 Division of Cancer Epidemiology and Genetics, National Cancer Institute, Bethesda, MD USA; 4 Kaiser Permanente Center for Health Research, Portland, OR USA; 5 College of Public Health, University of Iowa, Iowa City, IA USA; 6 Department of Radiology, University of California, San Francisco, CA USA; 7 KCJA, Luynes, France; 8 Feinberg School of Medicine, Northwestern University, Chicago, IL USA; 9 Louisiana State University Health Sciences Center, New Orleans, LA USA; 10 University of Hawaii Cancer Center, Honolulu, HI USA

**Keywords:** Breast cancer, Breast density, Puberty

## Abstract

**Background:**

Earlier age at onset of pubertal events and longer intervals between them (tempo) have been associated with increased breast cancer risk. It is unknown whether the timing and tempo of puberty are associated with adult breast density, which could mediate the increased risk.

**Methods:**

From 1988 to 1997, girls participating in the Dietary Intervention Study in Children (DISC) were clinically assessed annually between ages 8 and 17 years for Tanner stages of breast development (thelarche) and pubic hair (pubarche), and onset of menses (menarche) was self-reported. In 2006–2008, 182 participants then aged 25–29 years had their percent dense breast volume (%DBV) measured by magnetic resonance imaging. Multivariable, linear mixed-effects regression models adjusted for reproductive factors, demographics, and body size were used to evaluate associations of age and tempo of puberty events with %DBV.

**Results:**

The mean (standard deviation) and range of %DBV were 27.6 (20.5) and 0.2–86.1. Age at thelarche was negatively associated with %DBV (*p* trend = 0.04), while pubertal tempo between thelarche and menarche was positively associated with %DBV (*p* trend = 0.007). %DBV was 40% higher in women whose thelarche-to-menarche tempo was 2.9 years or longer (geometric mean (95%CI) = 21.8% (18.2–26.2%)) compared to women whose thelarche-to-menarche tempo was less than 1.6 years (geometric mean (95%CI) = 15.6% (13.9–17.5%)).

**Conclusions:**

Our results suggest that a slower pubertal tempo, i.e., greater number of months between thelarche and menarche, is associated with higher percent breast density in young women. Future research should examine whether breast density mediates the association between slower tempo and increased breast cancer risk.

## Background

**Fig. 1:**
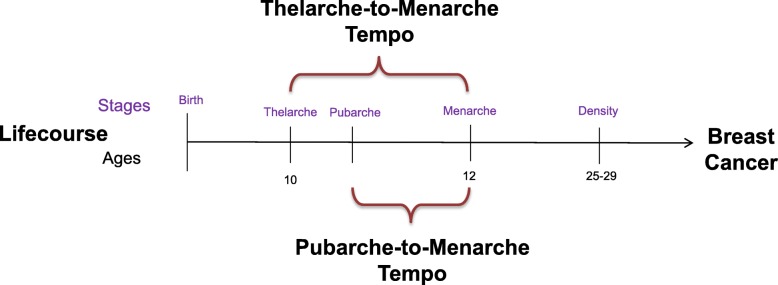
Pubertal and breast density risk factors for breast cancer across the life course. Thelarche and pubarche mark the onset of breast development and pubic hair growth, respectively. Menarche is the onset of menstruation. The age at onset determines the pubertal timing of these events, and the time between these events is known as pubertal tempo

An earlier age at menarche is an established risk factor for breast cancer [1–3]. However, menarche is only one relatively late part of the complex female pubertal transition, which also includes thelarche (the onset of breast development) and pubarche (the onset of pubic hair growth). Thelarche, typically the first sign of puberty, usually occurs 2–4 years before menarche [4]. An earlier recalled age at thelarche is associated with a 20% increased risk of breast cancer [5]. Pubertal tempo, that is the length of time between thelarche and menarche, also was positively associated with increased risk, independent of either age at thelarche or menarche [5]. Possible inaccurate recall of pubertal timing could attenuate the association with breast cancer, highlighting the need to assess both age and tempo of pubertal development prospectively in longitudinal studies. Yet connecting pubertal timing with a breast cancer diagnosis that occurs more than 50 years later is an obstacle for epidemiological studies [6]. One way to address this challenge is to assess intermediate markers that can be measured earlier in life. Breast density is one of the strongest predictors of premenopausal and postmenopausal breast cancer risk [7, 8] and may be a useful intermediate marker between early life factors, such as pubertal development, and breast cancer risk (see Fig. 1).

Characterizing the relationship between pubertal development and breast density may help clarify if breast cancer risk is influenced by factors during breast development, when breast tissue might be particularly susceptible to proliferative and carcinogenic stimuli. Given that earlier ages at thelarche and menarche are associated with increased breast cancer risk [5] and that breast density is positively associated with risk [9], we hypothesized that adult women who had an earlier thelarche and slower pubertal development would have denser breasts compared to those who had a later thelarche and faster pubertal development. We tested this hypothesis in the Dietary Intervention Study in Children (DISC) cohort, a prospective study originally designed as a diet intervention in children, who were re-visited in their mid-to-late twenties [10]. The objective of this investigation was to examine the relationship between prospectively assessed pubertal timing (age at thelarche, pubarche, and menarche) and tempo (the interval between thelarche and menarche or pubarche and menarche) with percent dense breast volume (%DBV) measured at ages 25–29 years.

## Methods

### Population

Between 1988 and 1997, DISC, a multicenter randomized controlled clinical trial sponsored by the National Heart, Lung, and Blood Institute (NHLBI), was conducted to test the safety and efficacy of a dietary intervention to reduce serum low-density lipoprotein cholesterol (LDL-C) in children with elevated LDL-C [10–12]. At baseline, 663 healthy, pre-pubertal, 8–10-year-old children, including 301 girls, with elevated LDL-C were recruited into DISC at six clinical centers and randomized to a behavioral dietary intervention or usual care control group. The intervention continued until the mean age of participants was 16.7 years. In 2006–2008 when participants were 25 to 29 years old, the DISC06 Follow-Up Study was conducted to evaluate the longer-term effects of the diet intervention on breast cancer risk factors in female participants. Prior to randomization, parents/guardians provided informed consent and DISC participants provided assent, while participants provided informed consent prior to the DISC06 follow-up visit. An NHLBI-appointed independent data and safety monitoring committee monitored the original DISC trial. Institutional review boards at all participating clinical centers and the data coordinating center approved both the DISC and the DISC06 Follow-Up Study protocols. The DISC06 Follow-Up Study protocol also was approved by the Fox Chase Cancer Center’s Institutional Review Board.

All female DISC participants were invited to participate in the DISC06 Follow-Up Study, and 260 (86.4%) of the 301 females originally randomized took part. Women who were pregnant or breastfeeding at or within 12 weeks before the study visit (*n* = 30) and those who had breast implants or breast reduction surgery (*n* = 16) were not eligible for the current analysis. An additional 32 women were excluded because they had technically unacceptable or missing breast magnetic resonance imaging (MRI) leaving a total of 182 participants for inclusion in analyses.

### Data collection

#### Pubertal staging

Trained and certified clinicians assessed girls for breast and pubic hair Tanner stage (T1–5) at annual visits between the ages of 8 and 17 years until T5 was reached [13]. Ages at thelarche and pubarche were calculated by taking the mid-point of age between the two visits when girls transitioned from T1 to T2+ for the appearance of breast development and pubic hair, respectively. Participants reported whether they had begun menstruating at each visit and if so, their age at menarche in years and months [13]. Age at thelarche was subtracted from age at menarche for each girl to create the thelarche-to-menarche tempo variable, and age at pubarche was subtracted from age at menarche to create the pubarche-to-menarche tempo variable.

#### Breast density measurement

%DBV was measured using noncontrast MRI on a whole-body 1.5-Tesla or higher field-strength MRI scanner with a dedicated breast-imaging radiofrequency coil as previously described [10, 14]. One investigator (Dr. C. Klifa at the University of California, San Francisco, San Francisco, CA) processed all MRI image data by identifying the chest wall–breast tissue boundary and skin surface and using automated fuzzy C-means to separate breast fibroglandular and fatty tissue [14]. These methods allow for the measurement of total breast volume and absolute dense breast volume (ADBV), which quantifies fibroglandular tissue. We calculated the absolute non-dense breast volume (ANDBV) by subtracting ADBV from total breast volume and %DBV as the ratio of ADBV to total breast volume multiplied by 100. We averaged the density measures of both breasts for analysis. %DBV was the primary outcome and is highly correlated with percent breast density (*r* = .87) based on mammography [15], which is an established risk factor for breast cancer [16]. ADBV and ANDBV were secondary outcomes.

#### Covariates

At DISC06 follow-up visits, participants completed several questionnaires on demographic characteristics; medical, reproductive, and menstrual histories; medication use; and health habits. Height, weight, and waist circumference were measured, and body composition was assessed by whole-body dual-energy X-ray absorptiometry (DXA) as previously described [10].

### Statistical analysis

We initially explored distributions of breast density measures graphically and by using nonparametric statistics, and prior to modeling, log-transformed measures to improve normality. We calculated geometric means and 95% confidence intervals of %DBV, ADBV, and ANDBV across quartiles of ages at thelarche, pubarche, menarche, and pubertal tempo variables by exponentiating the least square means from multivariable linear mixed-effects regression models with clinic as a random effect and robust standard errors. Model 1 is the crude, unadjusted model. Model 2 adjusted for adult covariates, including parity (nulliparous vs parous), duration of hormone use (continuous), education (some college or less, bachelor’s degree, graduate degree), race (white vs. non-white), smoking status (never vs ever), whole-body percent fat measured by DXA (continuous), and height (continuous). Because childhood BMI is a strong predictor of pubertal timing and was also previously associated with %DBV in DISC06 [17], model 3 adjusted for the same covariates in model 2 as well as BMI at 8–10 years of age, expressed as a *z*-score relative to CDC 2000 Growth Charts [18]. Missing values of whole-body percent fat (*N* = 6), age at thelarche (*n* = 12), and age at pubarche (*n* = 9) were imputed using values from a prediction model that included adult BMI as an independent variable as well as covariates in model 3; this process was repeated 25 times to create 25 multiply-imputed datasets. Results from each imputed dataset were pooled using Rubin’s rule [19]. Using model 3, we explored the combined effects of pubertal timing (ages at thelarche and menarche) and thelarche-menarche tempo with breast density. Each of the continuous pubertal timing variables was dichotomized at the median and cross-classified with the similarly dichotomized tempo variable creating two dummy variables each with four categories: (1) early menarche/short tempo (reference), early menarche/long tempo, late menarche/short tempo, late menarche/long tempo; and (2) early thelarche/short tempo (reference), early thelarche/long tempo, late thelarche/short tempo, late thelarche/long tempo. We tested for interactions between thelarche-to-menarche tempo and diet intervention assignment as well as between thelarche and menarche by including cross-product terms in the fully adjusted models. In sensitivity analyses, we tested if the observed associations with %DBV held in subsets restricted to white participants, nulliparous participants, women not using hormonal contraceptives, or women whose baseline BMI *z*-score was < 1.5. All tests were two-sided and considered to be significant if *p* value < 0.05. All analyses were conducted using STATA 13.0 (College Station, TX).

## Results

At baseline, all girls were pre-pubertal, and during the DISC trial, the majority reached thelarche before pubarche. The mean age of 182 women included in the present study was 27.2 years at the DISC06 follow-up visit (Table 1). The majority were white (90%), nulliparous (71%), and ever users of hormonal contraceptives (93%, with 58% current users). Their mean BMI was 25.4 kg/m^2^. The mean (standard deviation) of %DBV was 27.6 (20.5). Covariates across tempo categories were generally similar (shown in Additional file 1: Table S1). The three related pubertal milestones—menarche, thelarche, and pubarche, were moderately correlated; Pearson correlation coefficients ranged from *r* 0.42 to 0.46. The correlations between menarche and the tempo variables were higher (thelarche-to-menarche tempo *r* = 0.68; pubarche-to-menarche tempo *r* = 0.61).

**Table 1:** Characteristics of DISC participants in childhood and as young adults

	Number	Mean	SD	Percentage
Child characteristics				
Race/ethnicity				
White	164			90
Other	18			10
Age at baseline, years	182	9.13	0.59	
BMI *z*-score at baseline	182	0.23	0.90	
Age at thelarche, years	170	10.59	1.09	
Age at pubarche, years	173	10.97	1.19	
Age at menarche, years	182	12.90	1.26	
Adult characteristics				
Age at follow-up, years	182	27.17	1.02	
BMI, kg/m^2^	182	25.39	5.36	
DXA % body fat, %	176	35.41	8.82	
Exogenous hormone use				
Never	11			6
Former	66			36
Current	105			58
Duration of hormone use, years	171	5.6	3.5	
Parous (vs nulliparous)	53/182			29
Education				
Graduate degree	25			14
Bachelor’s degree	95			52
Some college or less	62			34
Ever smokers (vs never-smokers)	82/182			45
Breast density measures				
Percent dense breast volume (%)	182	27.6	20.5	
Absolute dense breast volume (cm^3^)	182	104.2	70.6	
Absolute non-dense breast volume (cm^3^)	182	413.3	364.3	

Table 2 shows associations of pubertal timing and tempo with adult %DBV. Across the three models, the thelarche-to-menarche tempo association was consistently associated with %DBV. Age at thelarche was associated with %DBV after adjustment for child BMI *z*-score, whereas the association with age at menarche was attenuated. Age at pubarche was not associated with %DBV in any model, and pubarche-to-menarche tempo was not associated with %DBV after adjustment for covariates. In fully adjusted models (model 3), %DBV increased with increasing duration from thelarche- to- menarche tempo (*p* trend = 0.007). %DBV was 40% higher in women whose thelarche-to-menarche tempo was 2.9 years or longer (geometric mean (95%CI) = 21.8% (18.2–26.2)) compared to women whose thelarche-to-menarche tempo was less than 1.6 years (geometric mean (95%CI) = 15.6% (14.2–20.7)). Girls who were oldest at thelarche (≥ 11.1 years) had an 18% lower %DBV compared to girls who were youngest (8.7 to < 9.9 years; *p* trend = 0.04).

**Table 2:** Geometric mean (95% confidence interval (CI)) from mixed-effect regression models for each pubertal factor in relation to percent dense breast volume (%DBV)

Pubertal characteristic	Model 1	Model 2	Model 3
Age at thelarche, years			
8.7 to < 9.9	19.56 (15.62–24.48)	18.74 (16.93–20.76)	20.83 (18.42–23.57)
9.9 to < 10.4	19.36 (14.15–26.48)	19.06 (16.24–22.36)	19.50 (16.36–23.24)
10.4 to < 11.1	16.27 (12.01–22.05)	18.36 (14.54–23.19)	17.79 (14.15–22.38)
11.1+	19.90 (12.41–31.91)	18.72 (13.55–25.86)	17.04 (13.28–21.86)
*p* trend	0.56	0.94	0.04
Age at pubarche, years			
8.6 to < 10.3	22.08 (18.18–26.81)	19.04 (16.9–21.45)	20.84 (18.2–23.86)
10.3 to < 10.9	17.31 (12.27–24.42)	16.88 (11.9–23.94)	16.97 (12.21–23.57)
10.9 to < 11.5	16.20 (13.84–18.98)	17.51 (14.91–20.56)	17.73 (14.81–21.23)
11.5+	19.72 (13.74–28.32)	21.66 (18.25–25.71)	19.47 (17.38–21.83)
*p* trend	0.87	0.39	0.64
Age at menarche, years			
10 to < 12.2	16.89 (13.04–21.87)	15.96 (13.28–19.19)	17.10 (14.15–20.66)
12.2 to < 12.8	14.38 (12.03–17.19)	15.39 (11.53–20.54)	16.38 (12.4–21.65)
12.8 to < 13.4	19.98 (15.19–26.27)	22.90 (20.37–25.75)	21.65 (19.36–24.21)
13.4+	25.96 (17.03–39.56)	21.76 (16.41–28.86)	20.18 (15.66–25.99)
*p* trend	0.03	0.01	0.13
Thelarche-to-menarche tempo, years			
< 1.6	13.90 (10.23–18.88)	15.45 (14.08–16.95)	15.55 (13.85–17.47)
1.6 to < 2.3	19.91 (16.02–24.76)	18.88 (16.17–22.04)	19.49 (16.89–22.5)
2.3 to < 2.9	18.90 (13.14–27.18)	18.47 (15.77–21.62)	18.38 (15.7–21.51)
2.9+	24.85 (16.51–37.40)	22.55 (17.8–28.56)	21.84 (18.18–26.24)
*p* trend	0.01	0.004	0.007
Pubarche-to-menarche tempo, years			
< 1.1	14.40 (10.03–20.66)	15.9 (12.23–20.68)	16.23 (12.3–21.41)
1.1 to < 1.7	17.16 (13.85–21.26)	18.69 (16.16–21.61)	18.40 (16.82–20.14)
1.7 to < 2.6	16.68 (11.20–24.85)	17.85 (13.7–23.25)	17.87 (13.88–23.02)
2.6+	29.09 (25.22–33.56)	22.80 (20.25–25.68)	22.66 (20.79–24.7)
*p* trend	< 0.001	0.06	0.06

Model 1 is unadjusted

Model 2 adjusts for the following variables as fixed effects: adult covariates, including parity (nulliparous vs parous), duration of hormone use (years, continuous), education (some college or less (reference), bachelor degree, graduate degree), race (white vs. non-white), smoking status (never vs ever), whole-body percent fat measured by DXA (%, continuous), and height (continuous). Clinic included as a random effect

Model 3 adjusts for the same factors in model 2, and in addition, BMI at 8–10 years of age expressed as a *z*-score relative to CDC 2000 Growth Charts (continuous)

There was a positive multiplicative interaction between thelarche and menarche in association with %DBV (*p* for interaction < 0.001).We, therefore, explored the combined effects of pubertal timing (age at thelarche and menarche) and thelarche-menarche tempo in cross-classified models and present the %DBV geometric means and 95% confidence intervals from these models in Table 3. Women with early menarche and short tempo had the lowest %DBV compared with women with early menarche and long tempo and women with late menarche and either short or long tempo. Women with early thelarche and short tempo had similar %DBV to women with either later thelarche and/or longer tempo (20.46% vs 19.81–20.4%) (15.73% vs. 18.84–21.33; *p* ≤ 0.01).

**Table 3:** Geometric mean (95% confidence interval (CI)) from mixed-effect regression models, stratified by median of thelarche, menarche, and thelarche-to-menarche tempo in relation to percent dense breast volume (%DBV)

	Number	Mean (95% confidence interval)	*p* value
Thelarche effect			
Early menarche/short tempo	62	15.73 (14.3–17.30)	Reference
Early menarche/long tempo	29	18.84 (17.05–20.81)	0.01
Late menarche/short tempo	29	21.33 (18.99–23.97)	< 0.001
Late menarche/long tempo	62	20.88 (17.49–24.92)	< 0.001
Menarche effect			
Early thelarche/short tempo	29	20.47 (16.18–25.91)	Reference
Early thelarche/long tempo	62	19.81 (17.49–22.44)	0.82
Late thelarche/short tempo	62	16.34 (14.64–18.23)	0.11
Late thelarche/long tempo	29	20.40 (16.52–25.20)	0.98

The means and 95% confidence intervals are generated from stratified models including the same covariates included in model 3 of Table 2. The following variables were adjusted as fixed effects: adult covariates, including parity (nulliparous vs parous), duration of hormone use (years, continuous), education (some college or less (reference), bachelor degree, graduate degree), race (white vs. non-white), smoking status (never vs ever), and whole-body percent fat measured by DXA (%, continuous), height (continuous), and BMI at 8–10 years of age expressed as a *z*-score relative to CDC 2000 Growth Charts. Clinic was adjusted for as a random effect

There was no interaction between thelarche-to-menarche tempo and diet intervention assignment. In sensitivity analyses, restricting to white women, nulliparous women, women not using hormonal contraceptives, or women with baseline BMI *z*-score < 1.5 did not change results substantially (data not shown).

Associations of pubertal timing and tempo with ADBV and ANDBV are shown in Additional file 1: Tables S2 and S3. None of the fully adjusted associations were statistically significant.

## Discussion

In this prospective study with over 20 years of follow-up, girls with a thelarche-to-menarche tempo of approximately 3 years or more have significantly higher %DBV in their mid-to-late twenties compared to girls with a tempo of ~1.5 years or less. Age at thelarche, but not menarche, also was associated with %DBV in our fully adjusted models.

This prospective study demonstrates an association between clinically assessed pubertal timing and tempo with %DBV in young women. One previous study by Schoemaker et al. evaluated recalled pubertal timing in relation to adult breast density [20]. In that study, which measured breast density from mammograms at age 40–75 years, women with higher absolute dense breast area had a longer thelarche-to-menarche duration independent of the timing of pubertal onset. The association of tempo with percent density followed a similar pattern but it was not statistically significant. The current study prospectively confirms a positive relationship between pubertal tempo with breast density, though not absolute dense breast volume. There were several differences between the previous study and ours. We measured volumetric breast density by MRI, whereas Schoemaker et al. measured areal breast density from mammograms, which could contribute to different results even though MRI and mammographic breast density measures are highly correlated [20]. We ascertained thelarche and pubarche by annual Tanner staging and menarche by self-report to the nearest month during adolescence, whereas in the previous study ages (in years) of thelarche and menarche were recalled decades later. We also measured height and weight in childhood, whereas participants in the study by Schoemaker et al. recalled their body size in relation to peers [20]. Furthermore, we adjusted for adult body fatness in our analysis using percent body fat from DXA as opposed to BMI. Lastly, differences in breast composition across the life course could alter associations with pubertal tempo. In particular, our participants were considerably younger and all were premenopausal at the time of breast density assessment, whereas 80% of participants in the study by Schoemaker et al. were postmenopausal [20]. In one other prospective study by Denholm et al., ages at thelarche and menarche were positively associated with percent breast water (which is positively associated with mammographic percent density) [21]. However, they did not directly asses the association between pubertal tempo and breast density.

The mean (SD) of %DBV in our study was 27.6 (20.5), which is slightly higher than that in another small study (*n* = 24) of healthy premenopausal Asian women by Chen et al. that found the mean %DBV to be 21.4 (8.4) [22]. In contrast, in a study of young women’s breast tissue composition by Boyd et al. [23], the median percent water was 45%, which is substantially larger than the percent dense breast tissue that we observed. Thicker MRI sections used in the Boyd study were more likely to contain mixtures of water and fat, which may have contributed to higher overall percent water values.

Earlier age at menarche is a long established risk factor for breast cancer [24]. Even though the average age at menarche stabilized around 12 years in the 1960s [25–27], there has been a continual rise in breast cancer incidence in women younger than 50 years old [28]. Several previous studies did not observe an association of age at menarche with breast density [29–31], while others found that later age of menarche is associated with higher density [20, 32–34]. Consistent with Shoemaker et al. [20], we show that the positive association of age at menarche and %DBV is attenuated after adjusting for childhood BMI. Alternatively, there is building evidence of the potential importance of age at breast development for predicting breast cancer risk [5]. Age at thelarche was inversely associated with non-dense breast area in ours and the study by Schoemaker et al. [20]. In another study of girls, Tanner breast stage was positively associated with concurrently measured adolescent breast density, and though attenuated, the association was still present after adjusting for menarcheal status [35]. The continual decline in age at thelarche [27, 36], the corresponding decrease in the correlation between age at menarche and age at thelarche over time [37], and the more recent finding that slow tempo is related to increased risk of breast cancer [5] suggest that the duration between thelarche and menarche may be at least as if not more informative of breast cancer risk than either marker of puberty alone.

Our study has limitations. To be eligible for the original DISC trial, children had to have high LDL-C defined as greater than or equal to the 80th and less than the 98th age- and sex-specific percentiles of the Lipid Research Clinics population, which for girls translates to 117.5–164.5 mg/dL [38]. Additional DISC eligibility criteria that girls be 7.8–10.1 years old and pre-pubertal (Tanner stage 1) may have excluded early as well as late maturers. Thus, our findings may not be generalizable to all healthy children and the resultant truncated distribution of pubertal milestones may have weakened observed associations. Furthermore, because early maturers tend to progress through puberty at a slower tempo [39], associations between tempo and density may have been weakened. While pubertal staging was assessed by a clinician, palpation was not used to distinguish between breast development and lipomastia. Therefore, breast development could have been overestimated in overweight girls. However, even after removing girls with high BMI *z*-scores (> 1.5), the pattern of associations between tempo and adult %DBV remained, suggesting that the finding is robust to any misclassification of lipomastia for breast development. Our findings suggest associations between age at thelarche and thelarche-to-menarche tempo and %DBV but cannot prove causation or rule out a common cause.

Despite these limitations, our study has several strengths. In particular, we were able to leverage prospectively collected data on pubertal development and breast density measured from the DISC study. Trained and certified personnel following standard protocols collected all data. Clinicians assessed sexual maturation and participants reported onset of menses annually. Finally, breast density was measured at age 25–29 years by MRI, which gives accurate and precise measurement of %DBV.

## Conclusions

Our finding that a longer pubertal tempo is associated with increased %DBV may help to explain the current increasing rates of early-onset breast cancer incidence [40]. In the last 50 years, there has been a secular decline in the age of thelarche but not menarche, which translates to pubertal tempo being elongated over time [25]. Now, puberty for the general population has come to look more like those women in our study with longer tempo and associated higher breast density. Longer tempo also has been associated with increased breast cancer risk [5]. Additional studies are needed to determine if breast density mediates the association between pubertal tempo and risk.

## Supplementary information

**Additional file 1 : Table S1.** Characteristics of DISC participants in childhood and as young adults by quartiles of thelarche to menarche tempo duration. **Table S2.** Geometric mean (95%CI) from mixed-effects regression models for each pubertal factor in relation to Absolute Dense Breast Volume (ADBV). **Table S3.** Geometric mean (95%CI) from mixed-effects regression models for each pubertal factor in relation to absolute non-dense volume (ANDBV).

## Abbreviations

ADBV: Absolute dense breast volume
ANDBV: Absolute non-dense breast volume
%DBV: Percent dense breast volume
LDL-C: Low-density lipoprotein cholesterol
DISC: Dietary Intervention Study in Children
T1–T5: Tanner stage
MRI: Magnetic resonance imaging
DXA: Dual-energy X-ray absorptiometry
