# Supplementary material for: Pubertal timing and breast density in young women: a prospective cohort study
Source: Breast Cancer Res. 2019 Nov 14;21:122. doi: 10.1186/s13058-019-1209-x (PMC6857297; doi:10.1186/s13058-019-1209-x)
Supplement: Supplementary file 1 — Additional file 1 : Table S1. Characteristics of DISC participants in childhood and as young adults by quartiles of thelarche to menarche tempo duration. Table S2. Geometric mean (95%CI) from mixed-effects regression models for each pubertal factor in relation to Absolute Dense Breast Volume (ADBV). Table S3. Geometric mean (95%CI) from mixed-effects regression models for each pubertal factor in relation to absolute non-dense volume (ANDBV). [file 13058_2019_1209_MOESM1_ESM.docx]

| Table S1: Characteristics of DISC participants in childhood and as young adults by quartiles of thelarche to menarche tempo duration. | | | | | | | | | | | | | | |  |  |  |
| --- | --- | --- | --- | --- | --- | --- | --- | --- | --- | --- | --- | --- | --- | --- | --- | --- | --- |
|  | Tempo Quartile 1 | | | | Tempo Quartile 2 | | | | Tempo Quartile 3 | | | | Tempo Quartile 4 | | | |  |
|  | N | Mean | SD | % | N | Mean | SD | % | N | Mean | SD | % | N | Mean | SD | % | P -values |
| Child Characteristics |  |  |  |  |  |  |  |  |  |  |  |  |  |  |  |  |  |
| Race/Ethnicity | 44 |  |  |  | 47 |  |  |  | 44 |  |  |  | 47 |  |  |  | 0.50 |
| White | 38 |  |  | 87% | 42 |  |  | 89% | 40 |  |  | 91% | 44 |  |  | 94% |  |
| Other | 6 |  |  | 13% | 5 |  |  | 11% | 4 |  |  | 9% | 3 |  |  | 6% |  |
| Age at baseline, years | 42 | 9.21 | 0.08 |  | 43 | 9.17 | 0.09 |  | 42 | 9.04 | 0.10 |  | 43 | 9.09 | 0.08 |  | 0.51 |
| BMI Z score at baseline | 42 | 0.34 | 0.14 |  | 43 | 0.37 | 0.12 |  | 42 | 0.14 | 0.13 |  | 43 | 0.07 | 0.14 |  | 0.33 |
| Age at thelarche, years | 42 | 11.09 | 0.16 |  | 43 | 10.74 | 0.16 |  | 42 | 10.05 | 0.12 |  | 43 | 10.25 | 0.15 |  | <0.001 |
| Age at pubarche, years | 42 | 10.91 | 0.16 |  | 42 | 10.80 | 0.14 |  | 42 | 10.62 | 0.17 |  | 41 | 11.29 | 0.20 |  | 0.05 |
| Age at menarche, years | 42 | 12.12 | 0.16 |  | 43 | 12.73 | 0.17 |  | 42 | 12.69 | 0.11 |  | 43 | 13.98 | 0.18 |  | <0.001 |
| Adult Characteristics |  |  |  |  |  |  |  |  |  |  |  |  |  |  |  |  |  |
| Age at follow-up, years | 42 | 27.33 | 0.15 |  | 43 | 27.27 | 0.16 |  | 42 | 27.13 | 0.16 |  | 43 | 26.94 | 0.14 |  | 0.28 |
| BMI, kg/m^2^ | 40 | 26.58 | 0.84 |  | 43 | 25.44 | 0.74 |  | 42 | 25.29 | 0.88 |  | 43 | 24.31 | 0.73 |  | 0.25 |
| DXA % Body fat, % | 42 | 38.51 | 1.17 |  | 43 | 35.28 | 1.38 |  | 40 | 35.30 | 1.75 |  | 41 | 34.92 | 1.34 |  | 0.25 |
| Exogenous hormone use | 42 |  |  |  | 43 |  |  |  | 42 |  |  |  | 43 |  |  |  | 0.51 |
| Never | 3 |  |  | 7% | 3 |  |  | 7% | 4 |  |  | 10% | 1 |  |  | 2% |  |
| Former | 14 |  |  | 33% | 12 |  |  | 28% | 19 |  |  | 45% | 16 |  |  | 37% |  |
| Current | 25 |  |  | 60% | 28 |  |  | 65% | 19 |  |  | 45% | 26 |  |  | 60% |  |
| Duration of hormone use, years | 42 | 6.20 | 0.54 |  |  | 5.16 | 0.57 |  |  | 5.07 | 0.60 |  |  | 4.67 | 0.48 |  | 0.23 |
| Parous (vs nulliparous) | 14/44 |  |  | 32% | 9/47 |  |  | 20% | 18/44 | |  | 42% | 11/47 |  |  | 24% | 0.07 |
| Education | 44 |  |  |  |  |  |  |  |  |  |  |  |  |  |  |  | 0.70 |
| Graduate Degree | 6 |  |  | 14% | 9 |  |  | 20% | 6 |  |  | 12% | 5 |  |  | 10% |  |
| Bachelors Degree | 26 |  |  | 59% | 23 |  |  | 49% | 22 |  |  | 51% | 23 |  |  | 49% |  |
| Some College or less | 12 |  |  | 27% | 15 |  |  | 31% | 16 |  |  | 37% | 19 |  |  | 40% |  |
| Ever smokers (vs never-smokers) | 20/44 |  |  | 46% | 18/47 |  |  | 40% | 18/44 | |  | 42% | 24/47 |  |  | 52% | 0.81 |
| Breast Density Measures |  |  |  |  |  |  |  |  |  |  |  |  |  |  |  |  |  |
| Percent dense breast volume (%) | 42 | 21.86 | 2.53 |  |  | 28.57 | 3.35 |  |  | 26.80 | 3.04 |  |  | 33.03 | 3.11 |  | 0.07 |
| Absolute dense breast volume (cm^3^) | 42 | 526.27 | 59.50 |  |  | 410.78 | 56.07 |  |  | 402.89 | 59.31 |  |  | 317.93 | 39.28 |  | 0.05 |
| Absolute nondense breast volume (cm^3^) | 42 | 91.14 | 8.91 |  |  | 111.93 | 11.22 |  |  | 93.60 | 9.47 |  |  | 118.98 | 12.02 |  | 0.17 |

| Table S2: Geometric mean (95%CI) from mixed-effects regression models for each pubertal factor in relation to Absolute Dense Breast Volume (ADBV | | | | | | | | | |
| --- | --- | --- | --- | --- | --- | --- | --- | --- | --- |
| Pubertal Characteristic | Model 1 | Model 2 | | | Model 3 | | | | |
| Age at thelarche, years | | | |  | | |  |  | |
| 8.7 to <9.9 | 80.44 (67.28-96.17) | | 75.21 (63.32-89.32) | | | 83.7 (71.16-98.46) | | |  |
| 9.9 to<10.4 | 76.56 (62.10-94.38) | | 76.69 (61.35-95.85) | | | 78.47 (61.36-100.36) | | |  |
| 10.4 to <11.1 | 82.58 (60.76-112.24) | | 87.2 (65.87-115.42) | | | 84.47 (63.42-112.52) | | |  |
| 11.1+ | 77.86 (57.15-106.07) | | 77 (57.62-102.9) | | | 70 (54.97-89.12) | | |  |
| p-trend | 0.34 | | 0.658 | | | 0.141 | | |  |
|  |  | |  | | |  | | |  |
| Age at pubarche, years |  | |  | | |  | | |  |
| 8.6 to <10.3 | 88.79 (67.94-116.03) | | 77.22 (59.89-99.56) | | | 84.96 (63.32-113.99) | | |  |
| 10.3 to <10.9 | 77.32 (52.36-114.18) | | 73.91 (51.42-106.23) | | | 74.3 (52.43-105.29) | | |  |
| 10.9 to <11.5 | 75.14 (60.52-93.28) | | 76.75 (63.22-93.18) | | | 77.79 (61.69-98.08) | | |  |
| 11.5+ | 80.90 (61.60-106.26) | | 87.86 (69.01-111.87) | | | 78.48 (63.04-97.69) | | |  |
| p-trend | 0.77 | | 0.508 | | | 0.734 | | |  |
|  |  | |  | | |  | | |  |
| Age at menarche, years |  | |  | | |  | | |  |
| 10 to <12.2 | 74.67 (66.12-84.32) | | 66.09 (63.13-69.19) | | | 71.33 (66.64-76.35) | | |  |
| 12.2 to <12.8 | 67.16 (51.03-88.40) | | 67.65 (51.6-88.68) | | | 72.5 (54.79-95.94) | | |  |
| 12.8 to <13.4 | 93.30 (78.17-111.36) | | 104.39 (94.27-115.6) | | | 98.07 (87.17-110.33) | | |  |
| 13.4+ | 87.24 (63.88-119.14) | | 82.3 (61.39-110.33) | | | 75.67 (57.71-99.21) | | |  |
| p-trend | 0.28 | | 0.009 | | | 0.277 | | |  |
|  |  | |  | | |  | | |  |
| Thelarche to Menarche Tempo, years |  | |  | | |  | | |  |
| <1.6 | 74.27 (60.42-91.30) | | 66.96 (59.96-74.77) | | | 67.44 (58.51-77.73) | | |  |
| 1.6 to 2.3 | 90.33 (63.48-128.55) | | 85.79 (65.38-112.56) | | | 88.81 (66.68-118.29) | | |  |
| 2.3 to <2.9 | 76.45 (56.87-102.77) | | 75.46 (59.29-96.05) | | | 75.08 (57.98-97.22) | | |  |
| 2.9+ | 90.63 (64.87-126.61) | | 88.61 (70.64-111.15) | | | 85.6 (71.56-102.4) | | |  |
| p-trend | 0.73 | | 0.141 | | | 0.244 | | |  |
|  |  | |  | | |  | | |  |
| Pubarche to Menarche Tempo |  | |  | | |  | | |  |
| <1.1 | 68.89 (53.05-89.46) | | 71.89 (54.07-95.57) | | | 73.46 (53.26-101.33) | | |  |
| 1.1 to <1.7 | 75.34 (61.49-92.32) | | 74.95 (60.37-93.05) | | | 73.75 (62.05-87.66) | | |  |
| 1.7 to <2.6 | 74.97 (49.34-113.91) | | 76.03 (53.93-107.19) | | | 76.15 (54.68-106.05) | | |  |
| 2.6+ | 100.40 (85.50-117.90) | | 93.18 (80.12-108.37) | | | 92.54 (83.15-103.01) | | |  |
| p-trend | 0.100 | | 0.206 | | | 0.239 | | |  |
|  |  | |  | | |  | | |  |
| Model 1 is unadjusted | | | | | | | | | |
| Model 2 adjusts for the following variables as fixed effects: adult covariates, including parity (nulliparous vs parous), duration of hormone use (years, continuous), education (some college or less, bachelor degree, graduate degree), race (white vs. non-white), smoking status (never vs ever), whole-body percent fat measured by DXA (%, continuous), and height (continuous).  Clinic was adjusted for as a random effect. | | | | | | | | | |
| Model 3 adjusts for the same factors in Model 2 and in addition, BMI at 8–10 years of age expressed as a z-score relative to CDC 2000 Growth Charts. | | | | | | | | | |

| Table S3: Geometric mean (95%CI) from mixed-effects regression models for each pubertal factor in relation to absolute non-dense volume (ANDBV) | | | | | |
| --- | --- | --- | --- | --- | --- |
| Pubertal Characteristic | Model 1 | Model 2 | Model 3 | | |
| Age at thelarche, years |  |  | | |  |
| 8.7 to <9.9 | 279.47 (223.62-349.28) | 278.16 (227.75-339.73) | | 270.36 (214.7-340.45) | |
| 9.9 to<10.4 | 274.78 (204.14-369.87) | 284.17 (238.6-338.43) | | 282.43 (238.79-334.05) | |
| 10.4 to <11.1 | 360.94 (310.24-419.91) | 322.41 (281.62-369.1) | | 325.16 (285.45-370.4) | |
| 11.1+ | 252.27 (178.04-357.45) | 273.61 (240.27-311.59) | | 280.61 (250.41-314.46) | |
| p-trend | 0.73 | 0.86 | | 0.58 | |
|  |  |  | |  | |
| Age at pubarche, years |  |  | |  | |
| 8.6 to <10.3 | 269.84 (212.44-342.76) | 284.62 (221.93-365.02) | | 278.71 (216.02-359.61) | |
| 10.3 to <10.9 | 305.89 (266.81-350.68) | 296.07 (264.41-331.53) | | 295.74 (264.99-330.06) | |
| 10.9 to <11.5 | 310.85 (244.28-395.58) | 310.3 (261.68-367.95) | | 309.39 (263.31-363.53) | |
| 11.5+ | 271.89 (210.84-350.62) | 266.72 (241.99-293.98) | | 273.4 (243.8-306.59) | |
| p-trend | 0.72 | 0.69 | | 0.99 | |
|  |  |  | |  | |
| Age at menarche, years |  |  | |  | |
| 10 to <12.2 | 309.32 (240.81-397.32) | 294.29 (241.41-358.76) | | 289.59 (236.55-354.52) | |
| 12.2 to <12.8 | 340.91 (284.96-407.85) | 312.5 (258.06-378.42) | | 307.96 (261.79-362.28) | |
| 12.8 to <13.4 | 323.36 (246.48-424.22) | 310.32 (275.71-349.27) | | 314.44 (279.89-353.26) | |
| 13.4+ | 201.52 (150.94-269.06) | 242.28 (212.44-276.32) | | 246.61 (221.46-274.62) | |
| p-trend | 0.02 | 0.17 | | 0.23 | |
|  |  |  | |  | |
| Thelarche to Menarche Tempo, years |  |  | |  | |
| <1.6 | 383.52 (285.71-514.80) | 306.35 (266.83-351.74) | | 305.89 (267.15-350.25) | |
| 1.6 to 2.3 | 288.18 (240.58-345.21) | 311.01 (259.11-373.3) | | 308.73 (260.59-365.77) | |
| 2.3 to <2.9 | 273.35 (200.02-373.56) | 289.09 (234.24-356.79) | | 289.4 (235.27-355.98) | |
| 2.9+ | 232.44 (156.65-344.91) | 254.73 (212.06-305.99) | | 256.61 (216.93-303.54) | |
| p-trend | 0.05 | 0.12 | | 0.11 | |
|  |  |  | |  | |
| Pubarche to Menarche Tempo |  |  | |  | |
| <1.1 | 359.28 (286.45-450.62) | 330.51 (289.3-377.59) | | 328.9 (292.88-369.35) | |
| 1.1 to <1.7 | 307.82 (257.58-367.88) | 267.96 (230.27-311.81) | | 268.93 (232.03-311.69) | |
| 1.7 to <2.6 | 304.64 (256.95-361.18) | 290.52 (256.7-328.8) | | 290.42 (257-328.19) | |
| 2.6+ | 211.49 (175.44-254.96) | 271.51 (241.48-305.28) | | 271.93 (242.36-305.11) | |
| p-trend | <0.001 | 0.11 | | 0.09 | |
|  |  |  | | |  |
| Model 1 is unadjusted |  |  | | |  |
| Model 2 adjusts for the following variables as fixed effects: adult covariates, including parity (nulliparous vs parous), duration of hormone use (years, continuous), education (some college or less, bachelor degree, graduate degree), race (white vs. non-white), smoking status (never vs ever), whole-body percent fat measured by DXA (%, continuous), and height (continuous).  Clinic was adjusted for as a random effect. | | | | | |
| Model 3 adjusts for the same factors in Model 2 and in addition, BMI at 8–10 years of age expressed as a z-score relative to CDC 2000 Growth Charts. | | | | | |
